# Paralytic Toxins Accumulation and Tissue Expression of α-Amylase and Lipase Genes in the Pacific Oyster *Crassostrea gigas* Fed with the Neurotoxic Dinoflagellate *Alexandrium catenella*

**DOI:** 10.3390/md10112519

**Published:** 2012-11-12

**Authors:** Jean-Luc Rolland, Kevin Pelletier, Estelle Masseret, Fabien Rieuvilleneuve, Veronique Savar, Adrien Santini, Zouher Amzil, Mohamed Laabir

**Affiliations:** 1 IFREMER, Université Montpellier 2, Centre National de la Recherche Scientifique, IRD, UM1, UMR 5119 “Ecologie des Systèmes Marins Côtiers”, Place E. Bataillon, CC93, 34095 Montpellier cedex 5, France; Email: pelletier-kevin@hotmail.fr (K.P.); estelle.masseret@univ-montp2.fr (E.M.); fabien.rieuvilleneuve@univ-montp2.fr (F.R.); adrien.santini@ifremer.fr (A.S.); mohamed.laabir@univ-montp2.fr (M.L.); 2 IFREMER, Département Environnement, Microbiologie et Phycotoxines (EMP/PHYC), Rue de l’Ile d’Yeu BP 21105 44311 Nantes CEDEX 3, France; Email: veronique.savar@ifremer.fr (V.S.); zouher.amzil@ifremer.fr (Z.A.)

**Keywords:** *Crassostrea gigas*, *Alexandrium catenella*, PSP toxins, digestion, gene expression

## Abstract

The pacific oyster *Crassostrea gigas* was experimentally exposed to the neurotoxic *Alexandrium catenella* and a non-producer of PSTs, *Alexandrium tamarense* (control algae), at concentrations corresponding to those observed during the blooming period. At fixed time intervals, from 0 to 48 h, we determined the clearance rate, the total filtered cells, the composition of the fecal ribbons, the profile of the PSP toxins and the variation of the expression of two α-amylase and triacylglecerol lipase precursor (TLP) genes through semi-quantitative RT-PCR. The results showed a significant decrease of the clearance rate of *C. gigas* fed with both *Alexandrium* species. However, from 29 to 48 h, the clearance rate and cell filtration activity increased only in oysters fed with *A. tamarense*. The toxin concentrations in the digestive gland rose above the sanitary threshold in less than 48 h of exposure and GTX6, a compound absent in *A. catenella* cells, accumulated. The α-amylase B gene expression level increased significantly in the time interval from 6 to 48 h in the digestive gland of oysters fed with *A. tamarense*, whereas the TLP gene transcript was significantly up-regulated in the digestive gland of oysters fed with the neurotoxic *A. catenella*. All together, these results suggest that the digestion capacity could be affected by PSP toxins.

## 1. Introduction

Harmful algal blooms (HAB) occur worldwide and have increased in frequency [1]. Since 1988, the French Atlantic coast (Brittany) has known proliferations of the dinoflagellate *Alexandrium minutum,* a PSP (Paralytic Shellfish Poisoning) toxin producer. *Alexandrium catenella*, a neurotoxic dinoflagellate, was observed for the first time in 1998 in the French Mediterranean waters, and has been responsible for major blooms (≥1 × 10^6^ cells/L), which developed during spring and/or autumn in the Thau lagoon. Oysters (*Crassostrea gigas*) and mussels (*Mytilus galloprovincialis*) cultivated in Thau have been frequently contaminated by PSP toxins and have shown toxicity exceeding the sanitary threshold of 80 µg STX equiv/100 g wet weight, which causes frequent farm closures resulting in economic losses. *Crassostrea gigas*, cultivated in the Thau lagoon with an annual production reaching 10,000 tons, is particularly exposed to *A. catenella* [2]. Within this context, it is necessary to understand the effects of PSP toxins on the digestive physiology and on the metabolic processes occurring in *C. gigas* tissues. Bivalves showed specific responses to HAB species [3]. For example, the exposure of *C. gigas* to the toxic *A. minutum* increased the frequency of microclosures of the valves [4]. Several toxic strains of *A. minutum*, *A. catenella* and *A. tamarense* have been shown to affect negatively the clearance rate of *C. gigas* which may reduce the accumulation of toxins in these bivalves [5,6,7,8]. While effects of the PSP toxins on the feeding rate of *C. gigas* have been studied, data on the effects of these toxins on the digestive processes of this mollusk, and particularly on the related enzymes, are scarce [9,10]. Among the digestive enzymes, α-amylases A and B are the most studied in oysters. The genes encoding α-amylase A and B enzymes were identified in *C. gigas* [11] and their molecular structure was characterized [12]. Their expression level was shown to be related to enzyme polymorphism [13] and activity in the digestive gland, and was in accordance with the digestive function of this organ [14]. The gene coding for α-amylase A was expressed at a high level and its expression increased (+18%) in digestive gland of oysters which were given a high food ration, whereas no change was observed in the expression of gene B coding for α-amylase B [11]. The triacylglycerol lipase enzyme plays an important role in fat hydrolysis. It preferentially splits the esters of long-chain fatty acids at positions 1 and 3, mainly producing 2-monoacylglycerol and free fatty acids, and it shows a considerably higher activity against insoluble emulsified substrates than against soluble ones. This gene transcription appears to be up-regulated under experimental hydrocarbon exposure [15]. Until now, no information was available on the expression of these genes in oysters exposed to neurotoxic dinoflagellates.

The main objective of the present study was to determine (i) the feeding activity, (ii) the kinetics of toxin accumulation and *de novo* produced toxins, and (iii) the expression of the key genes implied in the digestive processes encoding for α-amylase A and B, and for triacylglycerol lipase in *C. gigas* exposed to a toxic strain of *A. catenella* or a strain of *A. tamarense* non-producer of PSTs considered as the control.

## 2. Results and Discussion

### 2.1. Feeding Activity Parameters

#### Clearance Rate and Cell Filtration

**Figure 1:**
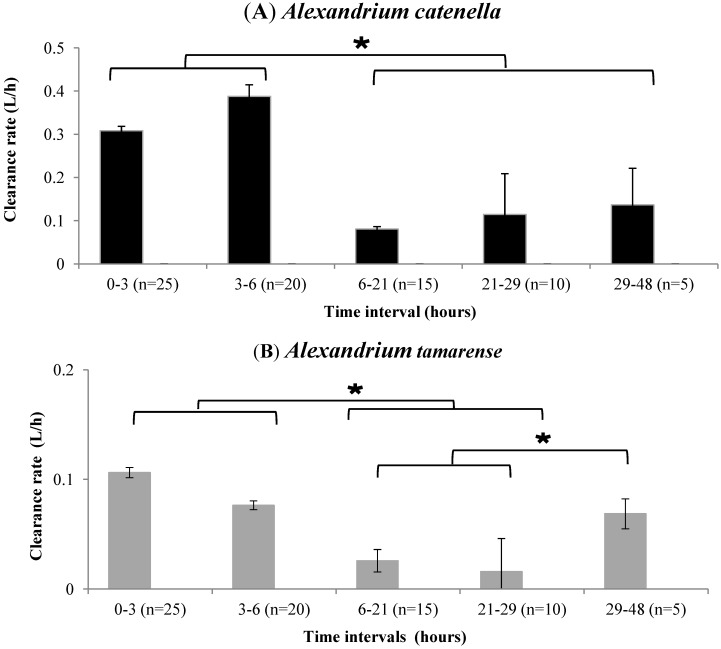
Variation of the clearance rate of *Crassostrea gigas* fed with *Alexandrium catenella* (**A**) and *Alexandrium tamarense* (**B**). (*n*) Number of oysters. * *p*<0.05.

No mortality of oysters occurred during any of the experiments. During the first 6 h of the experiments, the clearance rate (CR) was higher for oysters fed with *Alexandrium catenella* (0.308 ± 0.010 and 0.387 ± 0.022 L/h at 3 and 6 h, respectively) than for those fed with *Alexandrium tamarense* (0.106 ± 0.003 and 0.076 ± 0.057 L/h at 3 and 6 h, respectively) (Figure 1). After 21 h, the CR of oysters decreased significantly (*p* < 0.05) when they were fed with *A. catenella* (0.080 ± 0.056 L/h) or with *A. tamarense* (0.026 ± 0.007 at 21 L/h). From 29 h until the end of the experiments, the CR of oysters fed with *A. tamarense* increased significantly (0.068 ± 0.014 L/h) (*p* < 0.05) (Figure 1B). In contrast, the CR of oysters fed with *A. catenella* remained stable 0.114 ± 0.094 and 0.136 ± 0.085 L/h at 29 and 48 h (Figure 1A, respectively). 

**Figure 2:**
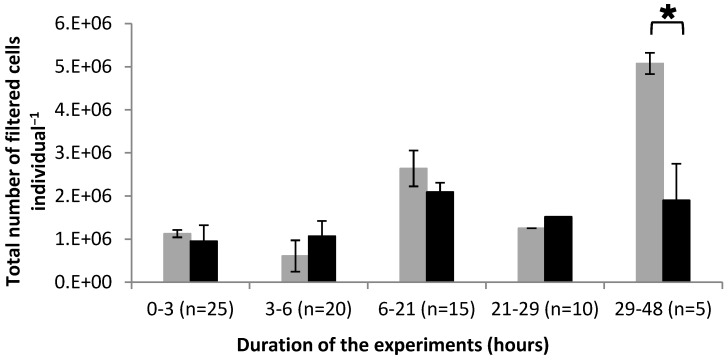
Variation of the total number of cells filtered by *Crassostrea gigas* fed with *Alexandrium tamarense* (**grey**) or with *Alexandrium catenella* (**black**). (*n*) Number of oysters in the tank. * *p*<0.05.

From 0 to 29 h, the CF was similar for oysters fed with *A. catenella* and for those fed with *A. tamarense* (Figure 2). These values ranged between 0.6 × 10^6^ ± 0.3 × 10^6^ cells/individual and 2.6 × 10^6^ ± 1.4 × 10^6^ cells/individual. From 29 to 48 h, the CF was significantly (*p* < 0.05) higher by around 170% for oysters fed with *A. tamarense* compared to that of oysters fed with *A. catenella* (Figure 2). The values were 1.9 × 10^6^ ± 0.8 × 10^6^ cells/individual and 5.1 × 10^6^ ± 0.2 × 10^6^ cells/individual for oysters fed with *A. catenella* and *A. tamarense*, respectively.

The data showed that the clearance rates (CR) measured between 0–3 h and 3–6 h of the feeding experiment were higher in *C. gigas* fed with *A. catenella* compared to those registered for oysters fed with *A. tamarense* (Figure 1). Such difference previously observed [6,7,16,17] could not be explained by morphological characteristics (size, shape) of the fed dinoflagellate species as they are quite similar [18]. We showed that the CR of *C. gigas* fed with both toxic and non-toxic *Alexandrium* species decreased between 6 and 29 h (Figure 1). This means that the toxicity of *A. catenella* was probably not the main factor explaining the decrease observed from 6 to 29 h. However, between 29 and 48 h, the CR of oysters fed with *A. catenella* did not change whereas that of oysters fed *A. tamarense* increased (Figure 1). It has been shown that some toxic strains of *Alexandrium minutum*, *A. catenella* and *A. tamarense* negatively affect the CR of *C. gigas* [5,7]. The CF of oysters fed with *A. catenella* cells did not increase from 21 h to the end of the experiment whereas it increased from 29 h when oysters were fed with *A. tamarense* (Figure 2). These data suggest that oysters were able to control their food uptake after a fixed time of exposure to the toxic alga which probably corresponds to a given toxin content in their tissue. 

### 2.2. Composition of Fecal Ribbons

**Figure 3:**
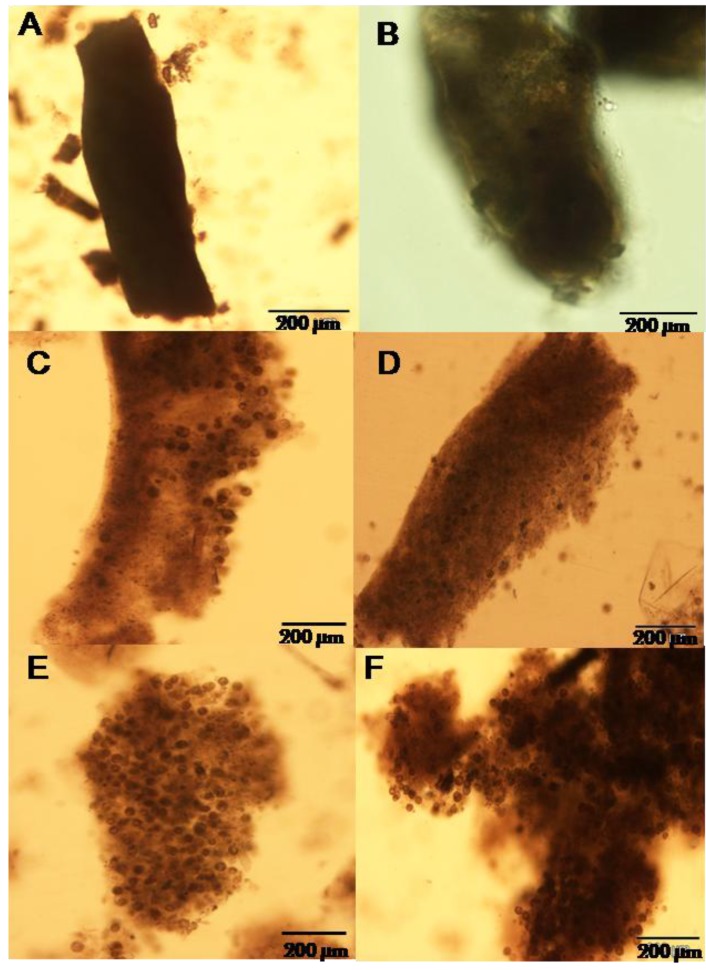
Light microscope photographs of feces produced by *Crassostrea gigas* fed with *Alexandrium catenella* (**A**, **C** and **E**) and with *Alexandrium tamarense* (**B**, **D** and **F**) during the feeding experiment.

Examination of feces after 3 h of the feeding experiment showed that the feces produced by *C. gigas* fed with either *A. catenella* or *A. tamarense* were composed mainly of digested cells (≥90%). (Figure 3A,B). From 6 h to 29 h, the percentage of intact cells in the feces of the two dinoflagellates started to increase and represented between 25% and 75% of the feces (Figure 3C,D). The undigested cells packed in the fecal ribbons correspond to pellicular cysts. The percentage of filling of the fecal ribbons by intact cells increased with the duration of the experiment and was maximal (≥90%) at 48 h (Figure 3E,F). 

As defecation is related to the digestive activity of oysters, these data suggest that the oysters quickly decreased its digestion activity. As several authors have previously shown [5,8], the toxicity of *A. catenella* should not be considered as the main factor for the explanation of the observed increase of intact cells produced in the feces, because the proportion of intact cells rejected in the feces was nearly the same in oysters fed with *A. tamarense* or *A. catenella*. However, the toxicity of the ingested cells could enhance the rejection capacity of oysters because it is a way for oyster to prevent the accumulation of toxins in its tissues. 

### 2.3. Toxin Profile and Concentration in the Algae and Fed Oysters

**Figure 4:**
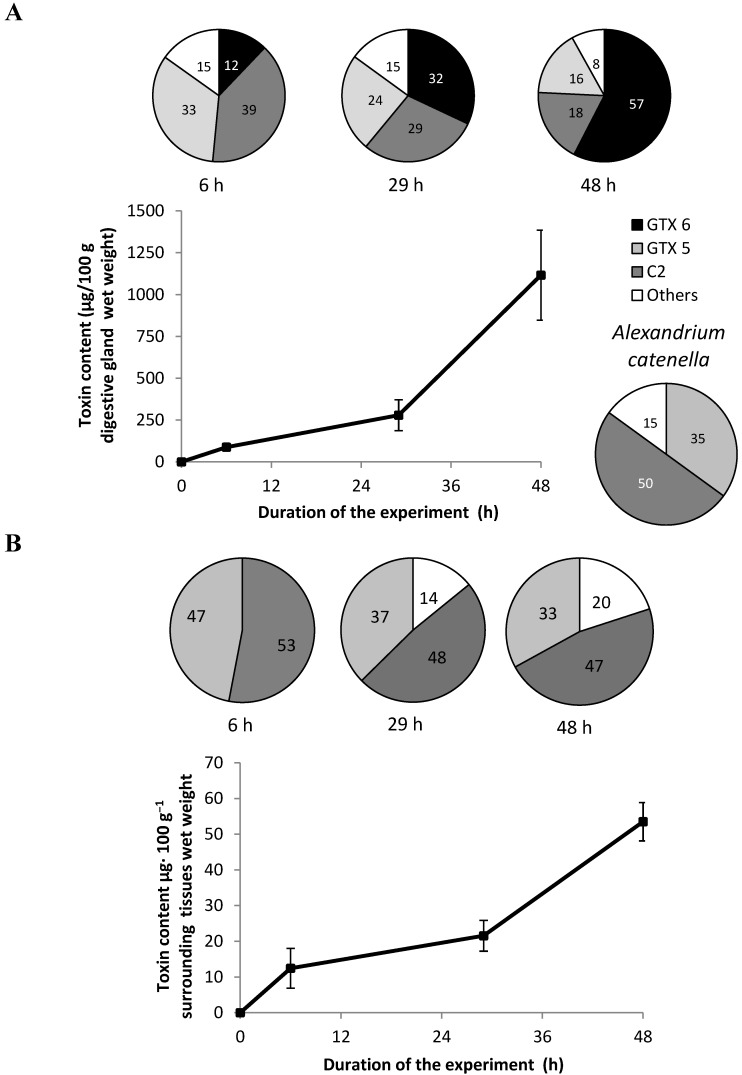
Evolution of the PSP-toxin content (µg STX equiv/100g wet weight) in digestive gland (**A**), and surrounding tissues (**B**) of *Crassostrea gigas* fed with *Alexandrium catenella*. Each Error bar shows the standard SD. The pie charts represent (in %) the temporal toxin composition.

The ACT03 strain contained 5.3 ± 0.4 pg toxins/cell. The specific toxicity of this strain was 1 ± 0.1 pg STX equiv/cell. The ACT03 toxin profile was characterized by the carbamate (STX, neo-STX, GTX1, 2, 3, 4 and 5), N-Sulfocarbamoyl (C2 and C4) and decarbamoyl (dc STX) toxins. The following toxins were found in decreasing concentrations: C2 (50%), GTX5 (35%), GTX4 (12%), Neo-STX (1%), GTX1 (1%), with GTX3, C4, STX and dcSTX present as trace amounts. No PSP toxins were detected in *Alexandrium tamarense* (ATT07 strain). During the 48-h experiment, PSP toxins accumulated in the digestive gland of oysters fed *Alexandrium catenella* (Figure 4A)*.* The toxicity level reached 85 (17), 241 (46) and 976 (156) µg/100 g tissue wet weight (µg STX equiv/100 g wet weight) at 6, 29 and 48 h, respectively. The toxicity level in the digestive gland of oysters quickly exceeded the sanitary threshold level (80 µg STX equiv/100 g tissue wet weight) at 29 h after the beginning of the experiment and onward. Moreover, the toxin profile of the digestive gland displayed some substantial change in comparison to that of *A. catenella* cells (Figure 4). The C2 and GTX5 content decreased from 50% and 35% to 18% and 16% of the total amount of toxins, respectively. The remaining toxins decreased from 15% to 8% and GTX6, which was absent in *A. catenella* cells represented 12% of the total toxins after six hours and was the major toxin compound (57%) in the digestive gland at the end of experiment. In the remaining tissues of oysters (Figure 4B), the toxicity level reached 12 (2), 21 (4) and 53 (9) µg. 100 g^−1^ tissue wet weight (µg STX equiv. 100 g^−1^ wet weight) at 6, 24 and 48 h, respectively. All the toxins, except GTX6, accumulated slightly in the remaining tissues of oysters, which could mean that there was a slight or no transformation in these tissues.

The data showed that the PSP toxins in the digestive gland of *Crassostrea gigas* increased rapidly and reached 156 µg STX equiv/100 g wet weight at 48 h, exceeding the sanitary threshold. The toxin concentration exceeding the sanitary threshold was not usually observed in the digestive gland of *C. gigas* fed with different toxic *Alexandrium* species [19]. In Mediterranean farm areas, *C. gigas* was characterized by toxin concentrations usually lower than that measured in other shellfish species and often remained under the sanitary threshold [20]. Moreover, the toxin concentration in the digestive gland of oysters fed experimentally with a toxic strain of *A. tamarense* was shown to approach the sanitary threshold [21]. This suggests that in the field, oysters may control the toxin level in their tissues to keep it as low as possible. In our experiments, *C. gigas* fed with *A. catenella* started to decrease its clearance rate at 21 h after the beginning of the feeding experiment with values of 279 (46) µg/100 g digestive gland wet weight (µg STX equiv/100 g wet weight) (Figure 4). However, this threshold could depend on the sensitivity of each individual and its capacity to transform and/or eliminate toxins, but could also be related to the toxin composition of the ingested algae. An important change in the toxin profile occurred in the digestive gland of *C. gigas* fed with the ACT03 strain. We noted a large decrease of the percentage of all toxins and particularly C2 and GTX 5, which were the most abundant toxins in the *Alexandrium* cells (Figure 4). GTX6, which was absent in the ACTO3 cells appeared in the digestive gland after 6 h of exposure and its concentration continually increased, reaching 57% of total amount of toxins after 48 h of exposure. It has been suggested that oysters implement a specific metabolic pathway transforming the accumulated PSP toxins into less toxic analogues and actively eliminate the toxins present in their tissues [21,22]. Differences between the toxin profiles of the ingested dinoflagellates and bivalve tissues have been attributed to a toxic-specific uptake, elimination, and metabolic interconversion of the accumulated toxins [23,24]. Several studies highlighted an active transformation of PSP toxins by epimerization between α and β-epimers, a reduction and an acid hydrolysis [23,25]. A model including the epimerization and reduction of PSP toxins provided a good description of the kinetics of the toxin accumulation and transformation in the blue mussel *Mytilus galloprovincialis* [26]. Unlike the study which showed that toxins could be transferred from the digestive gland to the other tissues in mollusk species [27], the present work showed that the GTX6 accumulated preferentially in the digestive gland and did not diffuse to the surrounding tissues of *C. gigas.* The accumulation in the digestive gland of *de novo* produced GTX6 (Figure 4) can result from the biotransformation/interconversion of the determined toxins (GTX5, C2 and others) and/or of non-identified compounds from the *A. catenella* cells. The ratio of the amount of toxins accumulated in the digestive gland of *C. gigas* relative to the amount of toxins available from the filtered *A. catenella* cells remained constant above 22% during the first 21 h of exposure then increased up to 38% at 48 h. This suggests that an important metabolic interconversion/elimination occurred in the *C. gigas* digestive gland. Hence, oysters could reduce the accumulation of toxins in their tissues by decreasing their filtration rate and stopping the digestion of already ingested cells, which were rejected intact in the feces (Figure 3). 

### 2.4. Temporal Expression of the Three Genes Related to Digestive Processes

**Figure 5:**
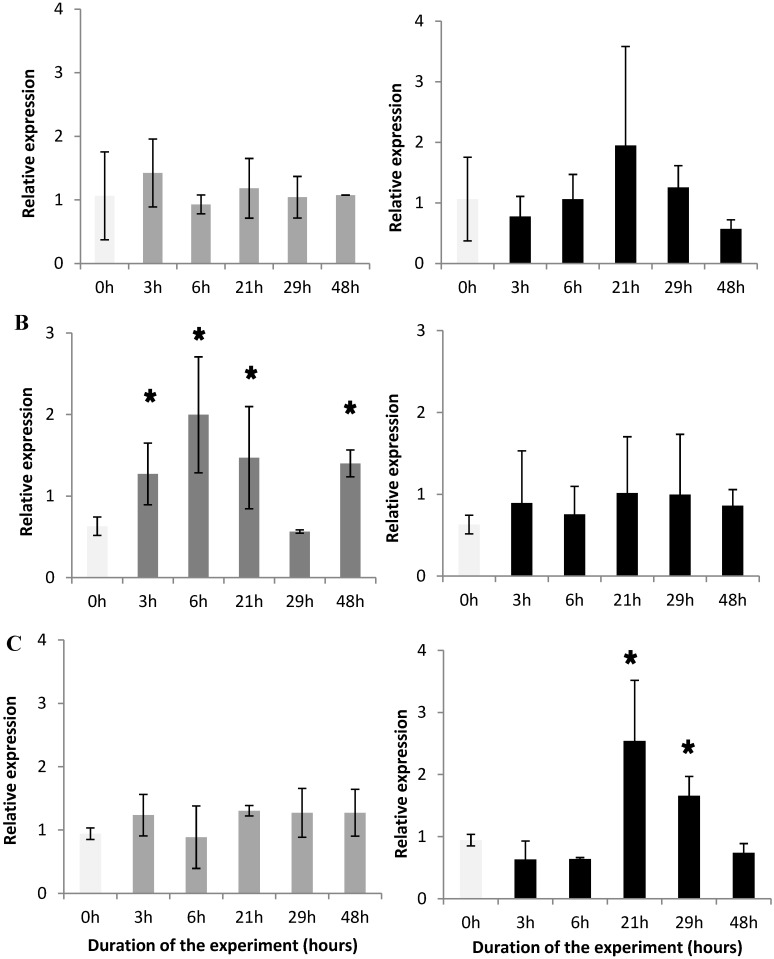
Amount of α-amylaseA transcripts (**A**), α-amylaseB transcripts (**B**), and triacylglycerol lipase precursor transcripts (**C**) in the digestive gland of the *Crassostrea gigas* fed with *Alexandrium tamarense* (**grey**), or with *Alexandrium catenella* (**black**) relative to the amount of transcripts in the digestive gland of the control unfed oysters (time 0, **light grey**). * *p*<0.05.

The analysis of the expression of the α-amylase gene A showed that this gene was not differentially expressed in the digestive gland of oysters fed with *Alexandrium catenella* or *Alexandrium tamarense* during the experiment(Figure 5A). In contrast, the α-amylase gene B was 50-fold less expressed than the α-amylase gene A in the digestive gland (not shown). The α-amylase gene B was significantly over-expressed (*P* < 0.05) from 3 h until the end of the experiment in oysters fed with *A. tamarense,* whereas this gene was not modulated in oysters fed with *A. catenella* (Figure 5B). Compared with the control, the level of the triacylglycerol lipase precursor gene transcript increased significantly (Figure 5C) in the digestive gland of oysters fed the toxic *A. catenella* (*P* < 0.05). It reached its highest level at 21 h after the beginning of the feeding experiment, then decreased gradually until 48 h. The TLP gene was not modulated in the digestive gland of oysters fed with the non-toxic *A. tamarense*.

Both α-amylases A and B transcripts were shown to be expressed predominantly in the digestive gland of *C. gigas* [11]. Here, we showed that the α-amylase A transcript expression, although it was important in the digestive gland, was not significantly modulated in the digestive gland of oysters fed with either toxic or non-toxic *Alexandrium* species. This result supports the hypothesis that the α-amylase A might be implicated in the hydrolysis of the glycogen stock in oysters instead of the newly ingested compounds [11]. For the first time, we observed an over-expression of the α-amylase B transcript in oysters fed with *A. tamarense*. This suggests that the promoter region and the putative regulatory elements previously identified could play a role in the modulation of the expression of the α-amylase B transcript [12]. Conversely, the induction of the expression of the α-amylase B transcript was not observed when oysters were fed with *A. catenella*. This toxic dinoflagellate seems to affect the α-amylase B transcript expression preventing the synthesis of the corresponding protein and consequently could affect the sugar digestive capacity of oysters. The functional role of the α-amylase B and its catalytic properties need to be further investigated. Lipases are mainly used by organisms for a range of metabolic functions including the assimilation and reorganization of dietary lipids and the mobilization of stored lipids [28]. The triacylglycerol lipase precursor transcript was demonstrated to be over-expressed in *C. gigas* in response to hydrocarbon exposure [15]. In the present study, the TLP transcript was up-regulated in *C. gigas* fed with the toxic *A. catenella* but not the non-toxic *A. tamarense*. This result suggests that the corresponding enzyme could play an important but not yet identified role in the digestive processes of oysters fed with the neurotoxic *A. catenella*.

## 3. Experimental Section

### 3.1. Biological Material

#### 3.1.1. Oysters

Adult Pacific oyster *Crassostrea gigas* were collected in November 2010 and November 2011 from an oyster farm in the Thau lagoon (Masson SARL, Languedoc-Roussillon, France) during periods when the water is cold and when blooms did not occur. The mean total oyster fresh weight was 13.0 ± 2.0 g, mean digestive gland weight was 0.6 ± 0.1 g and mean shell length was 93.0 ± 10.0 mm. Before the experiments, oysters were exposed to a continuous flow of filtered (10 µm) Mediterranean seawater maintained in partial starvation, having only bacteria and nanoplankton to feed, at a constant temperature of 20 ± 1 °C during two weeks for acclimatization.

#### 3.1.2. Microalgae

The experiments were carried out with a toxic strain of *Alexandrium catenella* (ACT03) and a non-producer of PSTs strain of *Alexandrium tamarense* (ATT07) isolated from the Thau lagoon in 2003 and 2007, respectively. Moreover, ATT07 strain has no negative affect on the biology of *Acartia margalefi*, a dominant calanoid copepod in Thau lagoon [29]. The ENSW (Enriched Natural Sea Water [30]) culture medium used was characterized by a salinity of 35 PSU. The two dinoflagellate species were cultivated in batch cultures and were grown at 20 ± 1 °C, under a cool-white fluorescent illumination (100 µmoles photons/m^2^/s) and a 12 h:12 h light:dark cycle. For the feeding experiments we used algae in their exponential growth phase.

### 3.2. Feeding Experiments

Two independent feeding experiments were carried out. For each experiment, after two weeks of acclimatization, 120 oysters were randomly placed in four tanks (30 individuals per tank) containing 10 liters of filtered (0.2 µm) seawater. The experiments were conducted at a constant temperature of 20 ± 1 °C. Cells of *Alexandrium catenella* (two experimental tanks) or *Alexandrium tamarense* (two control tanks) were added into tank water in concentrations corresponding to an *in situ* bloom in Thau lagoon [31]. The mean concentrations in tank water at the beginning of the experiments for toxic *A. catenella* and non-toxic *A. tamarense* were (1.35 ± 0.02) × 10^6^ cells/L and (2.20 ± 0.23) × 10^6^ cells/L, respectively. Fresh cells were regularly added at 3, 6, 21 and 29 h to approach the initial cell concentrations. However, during the 48-h experiments, the concentrations in tank water ranged between 1 × 10^6^ cells/L and 2.5 × 10^6^ cells/L. To estimate the concentration of cells in tanks during the experiment, triplicates of 1 mL of water were collected and cells were fixed with Formalin (2%) then counted in a Nageotte counting chamber using photonic microscope. 

### 3.3. Measurement of the Feeding Parameters

To determine the effects of the toxic *Alexandrium catenella* on the feeding behavior of *Crassostrea gigas*, clearance rate and cell filtration were measured during the feeding experiments.

#### 3.3.1. Clearance Rate

The clearance rate (CR) of oysters was measured according to the modified method of Coughlan’s [32]. In short, the oyster clearance rate was calculated using the following equation:

{CR = [Ln(N_i_/N_t_) M/t]/H} (1)

where N_i_ is the initial concentration of the microalgae in experimental tank water, N_t_ the concentration (cells/L) after t h, M the total volume of experimental water n the tank, t the duration time and H the number of oysters in tank.

#### 3.3.2. Cell Filtration

The number of filtered cells (CF) by each oyster individual was calculated using the following equation:

{CF = (N_i_ − N_f_) × V/H} (2)

where N_i_ is the initial concentration (cells/L) of microalgae in experimental tank, N_t_ the concentration (cells/L) after t h, V the volume of water in tank and H the number of oysters in tank.

### 3.4. Fecal Material Examination

At 3, 6, 21, 29 and 48 h following the beginning of the feeding experiment, all the feces produced were removed with a Pasteur glass pipette, pooled and stored in sea water with formalin (2%) until examination under an optical microscope. At least 15 feces were sorted randomly and examined for each time interval and for each experimental tank. The feces were observed by inverted microscope (Olympus) and photographed with a digital camera. The relative abundances as a percentage of digested (brown bulk material) and non-digested (intact and visible) cells in the feces were estimated.

### 3.5. Tissue Sampling

To determine the gene expression in the digestive gland of oysters and their PSP toxin content during the feeding experiment, 5 oysters were randomly taken from the tanks (containing each 30 individuals) at time 0 (control), 3, 6, 21, 29 and 48 h. One cubic millimeter of the digestive gland was collected and placed in 1 mL of Trizol buffer and conserved at −20 °C until RNA extraction. The remaining digestive glands and the other tissues were separately pooled and stored at −20 °C until the toxin extraction was performed. The gene expression analysis was conducted on individual digestive gland tissues.

### 3.6. Chemical Analysis by Liquid Chromatography/Fluorescence Detection (LC/FD)

1 mL of 0.1 N acetic acid was added to the pooled digestive gland or pooled remaining tissues and the samples were frozen at −20 °C until the extraction and analysis were performed. To release the toxins, the samples were sonicated for 5 min in a water bath three times, and centrifuged at 17,000 g for 10 min at 4 °C. The supernatants were used for the subsequent LC/FD PSP toxin analyses, using the method of Oshima (1995) [33]. The toxins were separated by reverse chromatography using a C_8_ column (5 μm Develosil, 4.6 mm i.d. × 250 mm) with a flow rate of 0.8 mL/min. The eluent pH and/or column temperature were calibrated to optimize the separation of some gonyautoxins (dc‑DTX3/B1/dc-GTX-2). The toxins were quantified using certified standards provided by CNRC Halifax-Canada. B2 and C2-toxins were detected and quantified indirectly after acid hydrolysis (HCl 0.4 N at 97 °C for 5 min) [2]. The toxin concentration (µg/g) was converted into µg STX equiv/100 g wet weight of tissues using the conversion factors determined by Oshima (1995) [23]. 

Triplicates of 10 mL batch cultures (cell concentration ≥ 10^7^ cells/L) were taken during the exponential growth phases of the cultivated dinoflagellates. After centrifugation (3000 g, 8 min, 4 °C), the cells were suspended in 1 mL of 0.1 N acetic acid and frozen at −20 °C. The extraction and toxin analyses were performed as explained above.

### 3.7. Semi-Quantitative Analyses of the Transcription of Digestive-Related Genes

The expression levels of the α-amylase A and α-amylase B genes, and triacylglycerol lipase precursor genes were measured in the digestive glands of *Crassostrea gigas* fed with *Alexandrium catenella* or *Alexandrium tamarense* at 0, 3, 6, 21, 29 and 48 h after the beginning of the experiment.

Total RNA was isolated from the oyster tissues using the standard Trizol method (Invitrogen, Carlsbad, California, USA), then treated with DNAse (Invitrogen) to eliminate contamination of genomic DNA. The DNAse was removed by phenol chloroform extraction and the quality and quantity of the extracted RNA was assessed by spectrophotometry at 260 nm. Subsequently, total RNA for each time of collection was pooled. Following heat denaturation (70 °C for 5 min), reverse transcription was performed using 1 μg of total RNA prepared with 50 ng/μL oligo-(dT)_12−18_ in a 20 μL reaction volume containing 1 mM dNTPs, 1 unit/μL of RNAseOUT and 200 units/μL MMLV reverse transcriptase in reverse transcriptase buffer according to the manufacturer’s instructions (Invitrogen).

Primer pairs, designed by Huvet [11], were used to quantify the expression level of the α-amylase genes. The primer pairs used to quantify the expression level of triacylglycerol lipase precursor were designed according to the sequence available in Gene-Bank. The expression of the ribosomal protein F40 was used as housekeeping gene control. The primer sequences and annealing temperature for the amplification are shown in Table 1. Real-time PCR amplifications were performed in the Light Cycler 480 (Roche). In short, the following components were mixed to the indicated end-concentration: 5 mM MgCl_2_, 0.5 μM of each primer, 2.5 μL of reaction mix (Light Cycler^®^ 480 SYBR Green I Master) in a final volume of 5 μL. Reverse transcribed RNA (1 μL) diluted 1/10 was added as the PCR template to the Light-Cycler master mix and the following run protocol was used: initial denaturation at 95 °C for 5 min; 95 °C for 10 s; 10 s annealing of primers at different temperatures depending on the primer pairs (see Table 1); 72 °C for 10 s with a single fluorescence measurement; melting curve program (65–97 °C) with a heating rate of 0.11 °C/s, a continuous fluorescence measurement and a cooling step to 40 °C. Each PCR was performed in triplicate.

For further expression level analysis, the crossing points (CP) were determined for each transcript using the Light Cycler software. The specificity of the real-time PCR product was analyzed on agarose gel and by melting curve analysis. The amount of α-amylase gene expressed was calculated relative to the amount of the ribosomal protein F40 housekeeping gene (because of its lower coefficient of variation) using the delta–delta Ct method [34].

**Table 1:** Primer sequences and annealing temperature (Tm) for amplification and size of the obtained products.

Gene	Primers sequences 5′→3′	Tm	Product size (bp)	Genbank ID
α-Amylase A	CAACGGGGACATGAGCATT	62	116	AF320688
	CGTTACGGAAGGCAACCA			
α-Amylase B	CGCGTCACGGACTTCATT	62	115	AF321515
	CAGCGTCATTGGAGTTAGGC			
Triacylglycerol lipase precursor	TCAAGGCCTGTGATTCTACC	60	96	CB617387
	CTCGGACGTCCATATCATCG			
Ribosomal protein F40 (RPL40)	AATCTTGCACCGTCATGCAG	60	149	FP004478
	AATCAATCTCTGCTGATCTGG			

### 3.8. Statistics

Data were analyzed using one-way ANOVA followed by Tukey test (Statistica 10.0 software) Values are mean ± SD of two independent experiments. * *p* < 0.05.

## 4. Conclusions

The toxin analyses in the tissues of oysters fed with *Alexandrium catenella* showed an important transformational activity in the digestive gland producing PSP toxins, mainly GTX6, which were not present in the ingested algae. The data also showed that oysters prevent toxin accumulation by modifying the clearance rate and stopping the digestion of the filtered cells, which explains the relatively low level of PSP contamination of *Crassostrea gigas* compared to other shellfish species, as reported in the literature. *C. gigas* modulated the gene expression of the studied digestive enzymes (α-amylase B and TLP) in relation to the ingestion of the PSP-producing *A. catenella*. Further studies on the expression of genes coding for other key enzymes implicated in the digestive and metabolic processes of this mollusk must be performed.
